# Retraction: PrP^ST^, a Soluble, Protease Resistant and Truncated PrP Form Features in the Pathogenesis of a Genetic Prion Disease

**DOI:** 10.1371/journal.pone.0316408

**Published:** 2024-12-19

**Authors:** The PLOS ONE Editors

Following the publication and subsequent correction of this article [1, 2], concerns were raised regarding results presented in Fig 2. Specifically,

- There appears to be a vertical discontinuity in the wt mouse 1 month panel of Fig 2C, between lanes 3 and 4.
- In Fig 2C, the following bands of the right TgMHu_2_ME199K/wt 3 month panel appear more similar than would be expected from independent results.
  ○ Lanes 6 and 8
  ○ Lanes 9 and 10

The similarity between the bands in lanes 9 and 10 was discussed in a previous correction for this article [2] which stated that the authors provided the original gel for a similar experiment for Fig 2C and that the concern was resolved. However, editorial reassessment of these data show that the data provided in support of the TgMHu_2_ME199K/wt 3-month results appear to match the published panel for the TgMHu_2_ME199K/wt 7-month panel instead.

During editorial follow-up of these concerns, the corresponding author provided underlying data for the 3-month panel which does not confirm the presence of the bands in lanes 7–10 of the published panel, affecting the interpretation of these results.

PLOS regrets that these issues were not identified during the previous correction of this article.

In light of the above concerns that question the reliability and integrity of the published results, the *PLOS ONE* Editors retract this article.

RG did not agree with the retraction. YFL, MM, KF, and OB either did not respond directly or could not be reached.
